# Significant improvement of apple (Malus domestica Borkh.) transgenic plant production by pre-transformation with a Baby boom transcription factor.

**DOI:** 10.1093/hr/uhab014

**Published:** 2022-01-28

**Authors:** Jiajing Chen, Sumathi Tomes, Andrew P Gleave, Wendy Hall, Zhiwei Luo, Juan Xu, Jia-Long Yao

**Affiliations:** 1 The New Zealand Institute for Plant and Food Research Limited, Private Bag 92169, Auckland 1142, New Zealand; 2 Key Laboratory of Horticultural Plant Biology (Ministry of Education), College of Horticulture and Forestry, Huazhong Agricultural University, 1 Shizishan Street, Wuhan, 430070, China; 3 Zhengzhou Fruit Research Institute, Chinese Academy of Agricultural Sciences, 32 Gangwan Road Zhengzhou 450009, China

## Abstract

BABY BOOM (BBM) is a member of the APETALA2/ETHYLENE RESPONSE FACTOR (AP2/ERF) family and its expression has been shown to improve herbaceous plant transformation and regeneration. However, this improvement has not been shown clearly for tree species. This study demonstrated that the efficiency of transgenic apple (Malus domestica Borkh.) plant production was dramatically increased by ectopic expression of the *MdBBM1* gene. “Royal Gala” apple plants were first transformed with a CaMV*35S-MdBBM1* construct (*MBM*) under kanamycin selection. These *MBM* transgenic plants exhibited enhanced shoot regeneration from leaf explants on tissue culture media, with most plants displaying a close-to-normal phenotype compared with CaMV*35S-GUS* transgenic plants when grown under greenhouse conditions, the exception being that some plants had slightly curly leaves. Thin leaf sections revealed the *MBM* plants produced more cells than the *GUS* plants, indicating that ectopic-expression of *MdBBM1* enhanced cell division. Transcriptome analysis showed that mRNA levels for cell division activators and repressors linked to hormone (auxin, cytokinin and brassinosteroid) signalling pathways were enhanced and reduced, respectively, in the *MBM* plants compared with the *GUS* plants. Plants of eight independent *MBM* lines were compared with the *GUS* plants by re-transforming them with an herbicide-resistant gene construct. The number of transgenic plants produced per 100 leaf explants was 0–3% for the *GUS* plants, 3–8% for five *MBM* lines, and 20–30% for three *MBM* lines. Our results provided a solution for overcoming the barriers to transgenic plant production in apple, and possibly in other trees.

## Introduction

Most tree species are highly recalcitrant to genetic transformation and the regeneration of transgenic plants. For example, the transformation of fruit tree species in the Rosaceae family remains relatively inefficient, despite many researchers having worked on improving these systems over several decades [1–8]. The Rosaceae plant family includes the economically important fruit tree crops, apple (*M. domestica*), pear (Pyrus communis L.), peach (Prunus persica (L.) Batsch), cherry (Prunus avium L.) and plum (Prunus domestica L.), with apple being the first of these fruit tree species to be transformed, in the 1980s^1^. The transformation efficiency for apple is still relatively low (0–3%) after numerous studies to make improvements [2–4]. Pear [5], cherry [6], and plum [7] have a transformation efficiency comparable to or even lower than that of apple. Peach is the most difficult of the aforementioned species to transform, with only transgenic roots being produced through the use of *Agrobacterium rhizogenes*^8^. This lack of efficient transformation systems in these important tree fruit crops is a severe impediment to our ability to carry out functional genomics studies and trait improvement.

In general, the low frequency of shoot regeneration is the major barrier to fruit tree transformation. It is well known that shoot regeneration is dependent on cell division and cell response to plant hormones, especially auxin and cytokinin responses [9, 10]. Genotypes of a species can differ widely in their response to plant hormones for shoot regeneration *in vitro*, and research to identify ideal genotypes with optimum response to hormone-induced shoot regeneration is both laborious and time consuming, and often fails. An alternative to identifying plant genotypes most amenable to shoot regeneration, is to genetically manipulate selected genotypes to enhance shoot regeneration by regulating the expression of a number of genes encoding transcription factors, cell cycle proteins, and proteins of hormone biosynthesis and signalling transduction [11–14].

The *BBM*, encoding an AP2/ERF transcription factor, is one the genes shown to promote plant regeneration. BBM has diverse functions in regulating cell proliferation, plant growth and development [14, 15]. Previous reports have shown that overexpression of native and heterologous *BBM* genes enhances shoot regeneration [14, 16–18]. *BnBBM* from Brassica napus was shown to enhance cell proliferation and regeneration capacity when ectopically overexpressed in Arabidopsis thaliana and *B. napus* L. [14]. The ectopic expression of *AtBBM* and *BnBBM* genes in tobacco (Nicotiana tabacum L.) activated cell proliferation and shoot regeneration [17]. *Arabidopsis thaliana* transgenic lines over-expressing *RcBBM1* and *RcBBM2* of Rosa canina L. exhibited enhanced shoot regeneration capacity in tissue culture [18]. In addition, overexpression of the *BBM* gene has been shown to directly improve transformation efficiency in plants. The ectopic expression of *BnBBM* enhanced the regeneration of transgenic plants of sweet pepper (Capsicum annuum L.) [19]. Furthermore, overexpression of *BBM* and *WUS2* genes together was shown to markedly increase transformation efficiency in maize (Zea mays L.), rice (Oryza sativa L.), sorghum (Sorghum bicolor L.) and sugarcane (Saccharum officinarum L.) [13]. More recently a study has shown that overexpression of the *ZmBBM2* gene of maize increased the transformation efficiency of two maize inbred lines from 3–6% to 20–21% [20].

Although overexpression of *BBM* could enhance plant transformation and regeneration, the resulting transgenic plants usually show aberrant phenotypes, such as compact architecture, rumpling leaf and infertility [21]. To overcome the problem of aberrant phenotypes, at least two methodologies have been used. The first is to regulate the subcellular localization of the BBM protein. As a transcription factor, BBM must be located in the nucleus to regulate the expression of its target genes. When BBM is fused to the glucocorticoid receptor (GR) steroid-binding domain, the BBM-GR fusion protein is localized in the cytoplasm, but can be re-located into the nucleus by treating the cells with the synthetic steroid dexamethasone (DEX) [16]. Using this approach, the function of the over-expressed BBM can be restricted to the transformation and regeneration stages in tissue culture by the presence of DEX. Using this steroid-inducible BBM system, transgenic plants without aberrant phenotypes were produced for Arabidopsis and sweet pepper [16, 19]. The second method is to use an inducible site-specific recombination system to remove the overexpressed *BBM* transgene after the *in vitro* transformation and regeneration stages. With a heat-shock inducible FRT/FLP site-specific recombination system, healthy transgenic poplar (Populus tomentosa Carr.) plants were produced [22]. Similarly a drought-inducible Cre/LoxP system was used to lessen the adverse impact of *ZmBBM* over-expression in transgenic maize, sorghum, rice and sugarcane plant [13].

It is clear that BBM can be used to enhance the efficiency of transgenic plant regeneration from herbaceous model and crop plants, and poplar trees. However, this effective enhancement by BBM in transformation and shoot regeneration of fruit tree crops has not been demonstrated. In this study, we showed that overexpression of *MdBBM1* can dramatically improve apple transformation efficiency. The results demonstrate the utility of BBM to overcome the transformation barriers in apple and perhaps other recalcitrant tree species.

## Results

### Identification of *BBM* genes in apple

Two apple *BBM* genes were identified by searching four apple reference genomes, “Golden Delicious” [23, 24], “Hanfu” [25] and “Royal Gala” (Yao et al, unpublished) (Table S1 and Fig. S1). The two genes are hereafter referred to as *MdBBM1* represented by MDP0000871080 located on chromosome 11, and MdBBM2 represented by MD04G1247700 and MDP0000125317 located on chromosome 4. The protein sequences of MdBBM1 and MdBBM2 were grouped together with BBMs from other plant species (Fig. S2a). MdBBM1 and MdBBM2 contained the BBM motifs in addition to AP2 and euANT domains (Fig. S2b). Five other apple proteins most closely related to the two MdBBMs contained the AP2 and euANT domains, but lacked BBM motifs, indicating there are only two *BBM* genes in these apple reference genomes. Previously reported *BBM* genes from multiple plant species contain nine exons, whereas the apple BBM gene models predict only eight exons, as shown by aligning their CDS sequences and genomic DNA sequences (Fig. S2c). The smallest exon of nine nucleotides (GTTTATCTG) encoding three amino acids VYL within the first AP2 domain was not predicted in the gene models, but is present in apple genomic DNA (Fig. S3). The presence of VYL in MdBBM proteins is consistent with the BBM sequences of other plant species (Fig. S3). Therefore, this exon should be added to the gene model and we included it when designing gene constructs for apple transformation.

**Figure 1:**
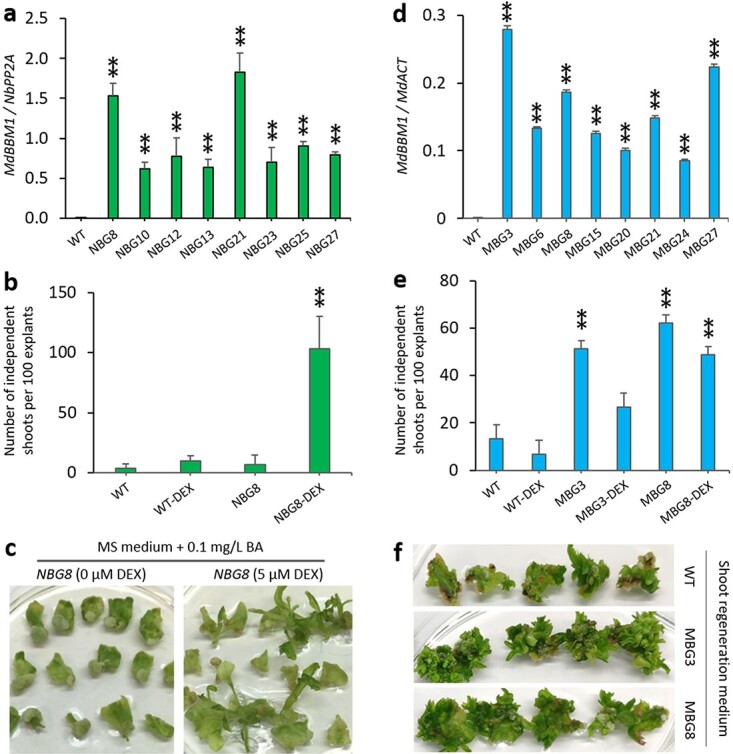
Analysis of tobacco and apple plants transformed with a 35S-MdBBM1-GR construct. a Relative transcript levels of MdBBM1 in wild-type (WT) and eight 35S-MdBBM1-GR transgenic lines (NBGs) of tobacco by ddPCR analysis. b Regeneration rates (number of independent shoots regenerated per 100 explants) for the WT and NBG8 line of tobacco on MS medium supplemented with 0.1 mg/L BA, without or with (+) DEX (5 μM). c Images show shoot regeneration from leaf explants of the WT and NBG8 on MS medium supplemented with 0.1 mg/L BA, with or without DEX (5 μM). d Relative transcript levels of MdBBM1 in WT and eight 35S-MdBBM1-GR transgenic lines (MBGs) of apple. e Regeneration rates for the WT, MBG3 and MBG8 line of apple on shoot maintenance medium with 0.1 mg/L IBA and 1 mg/L BA, without or with (+) DEX (5 mM). f Images show shoot regeneration from leaf explants of the WT, MBG3 and MBG8 line of apple on shoot regeneration medium with 1 mg/L NAA, 5 mg/L BA and 1 mg/L TDZ, without DEX. The values in a, b, d and e are mean ± SD (n = 3). The asterisks in a, b, d and e indicate significant differences to the WT according to a t-test (P < 0.01).

### *MdBBM1* overexpression enhanced apple shoot regeneration

As previous reports showed constitutive overexpression of *BBM* might induce sterility in the transgenic plants [13, 17], we generated a *35S-MdBBM-GR* construct to avoid the detrimental phenotype caused by constitutive overexpression of *BBM*. With this construct, the mRNA would be constitutively overexpressed, but the resulting fusion protein (BBM-GR) would be restricted to the cytoplasm and absent from the nucleus. Upon the application of dexamethasone (DEX), the BBM-GR fusion protein should be capable of relocating from the cytoplasm to the nucleus and thereby regulate the transcription of its target genes [16]. To test whether this construct worked as expected, we produced 27 transgenic tobacco lines (*NBG1–27*) with the *35S-MdBBM-GR* construct. We detected overexpression of *MdBBM1* mRNA in leaves of eight randomly selected transgenic tobacco lines compared with wild-type (WT) plants (Fig. 1a). Leaf explants of the WT tobacco plants showed low shoot regeneration rate, 3–10%, on the MS medium with a low concentration of cytokinin (0.1 mg/L BA) supplemented with 0 or 5 mM DEX, in three experiments (Fig. 1b). In the same experiments, leaf explants of *NBG8* showed a low shoot regeneration rate, 7%, without DEX (Fig. 1b). However, the addition of 5 μM DEX significantly increased the shoot regeneration rate of the *NGR8* transgenic line to 103% (Fig. 1b, c). This result indicated that the *35S-MdBBM-GR* construct functioned as expected in tobacco and that the *MdBBM1* had the ability to enhance tobacco shoot regeneration.

Using the *35S-MdBBM-GR* construct, we produced 29 independent transgenic apple plant lines (*MBG1–29*). We detected overexpression of *MdBBM1* mRNA in leaves of eight randomly selected transgenic apple lines compared with WT plants (Fig. 1d). The apple *MBG* lines, such as *MBG3* and *MBG8*, showed a medium rate of enhancement of shoot regeneration compared with the WT control on the shoot maintenance medium containing a sub-optimum level of cytokinin (1 mg/L BA) and without the inducer DEX (Fig. 1e). However, this enhancement was not further increased by the addition of the inducer (5 μM DEX) (Fig. 1e). We further tested these *MBG* plant lines on the shoot regeneration medium containing an optimum amount of cytokinin (5 mg/L BA +1 mg/L TDZ). An enhancement of shoot regeneration was observed for the transgenic lines in medium without the inducer DEX (Fig. 1f); however, the addition of the DEX inducer did not enhance shoot regeneration further. As many shoots were regenerated per leaf explant and these shoots were multiplying on the shoot regeneration medium, it was not feasible to count accurately the number of independently regenerated shoots. The regeneration rate was assessed visually only, but was clearly enhanced in the *MBG* lines tested (Fig. 1f). This result indicated that the medium rate of enhancement of shoot regeneration might be a consequence of the MdBBM1-GR fusion protein being able to enter the nucleus in the absence of the DEX inducer. This suggests that in apple there is a degree of “leakiness” of the GR element’s ability to sequester the fusion protein into the cytoplasm and that the fusion protein can enter the nucleus without the requirement for the DEX inducer.

As the DEX-induced relocation of the BBM-GR fusion protein did not work in apple, we further analysed the effect *MdBBM1* gene in apple using a *35S-MdBBM1* construct. We produced 29 independent transgenic apple plant lines with the *35S-MdBBM1* construct, and named the transgenic plants lines *MBM1–29*. We also produced ten independent transgenic apple plant lines with a *35S-GUS* gene construct as a control. Overexpression of *MdBBM1* mRNA was detected in eight randomly selected *MBM* lines compared with a *GUS* line (Fig. S4).

**Figure 2:**
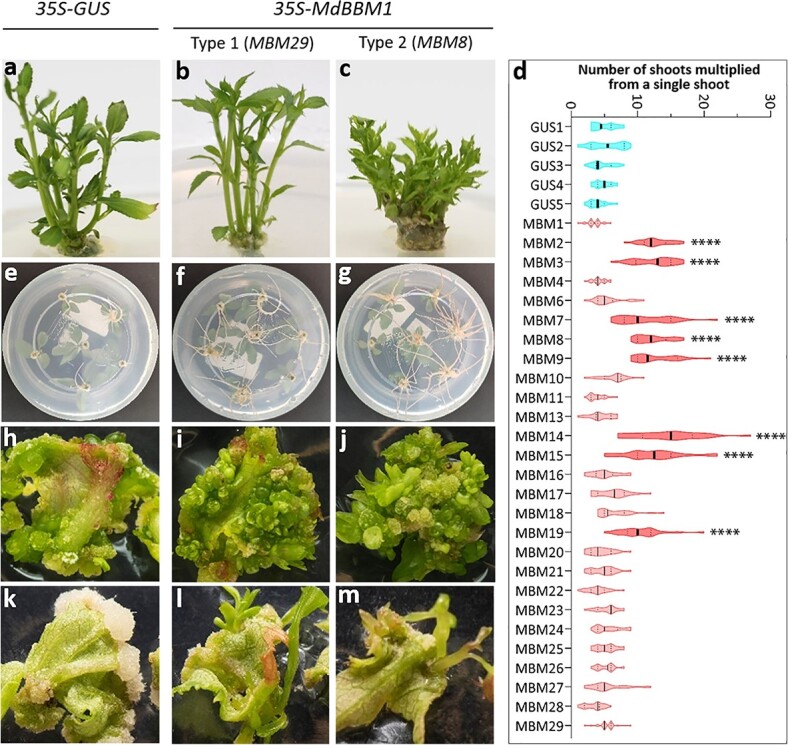
In vitro phenotype analyses of apple transgenic lines overexpressing MdBBM1. a–c Shoots multiplied from a single shoot of a 35S-GUS line and two types of 35S-MdBBM1 lines (types-1 MBM29 and type-2 MBM8) respectively, photographed after a single shoot was cultured on shoot maintenance medium for three weeks. d Number of shoots multiplied from each single shoot of 35S-GUS and 35S-MdBBM1 lines presented as violin-type plots. The data were collected from three independent experiments each used six to eight shoots per line. A one-way ANOVA test revealed the significant differences in shoot number between the GUS and MBM lines (**** representing p < 0.0001). e–g Root growth of the three lines at three weeks after six single shoots were cultured on rooting medium. h–j Shoot regeneration of the three lines at six weeks after leaf explants were culture on a shoot regeneration medium. k–m Shoot regeneration at six weeks after leaf explants were cultured on shoot maintenance medium. For shoot regeneration (h–m), the leaf explants were cultured for four weeks in the dark and then two weeks under a 16-h photoperiod.

We investigated the phenotypic differences between *in vitro* plantlets of the *MBM* lines and the *GUS* lines. The *MBM* lines showed two types of shoot architecture, which we named type-1 and type-2 (Fig. 2). On shoot maintenance medium, type-1 *MBM* shoots showed a similar branch number and plant architecture to those of the *GUS* shoots (Fig. 2a, b), while the type-2 *MBM* shoots were compact and produced more branches (Fig. 2c). Of the 29 *MBM* transgenic lines, eight (*MBM2–3*, *MBM7–9*, *MBM14–15*, and *MBM19*) had type-2 *MBM* shoots, while the other 21 belong to the type-1 *MBM* shoots category (Fig. 2d). The type-2 transgenic lines produced significantly more branches (10–15 branches per shoot) than the *GUS* and type-1 transgenic lines (approximate 5 branches per shoot) (one-way ANOVA analysis; *p* < 0.0001; Fig. 2d). Root development and morphology were also affected in the *MBM* transgenic lines. After 3 weeks *in vitro* culture on rooting medium, the *MBM* lines, especially type-2 lines, produced more roots than the *GUS* lines (Fig. 2e-g).

To determine the effect of the *35S*-*MdBBM1* construct on apple shoot regeneration, experiments were performed using leaf explants of *GUS*, type-1 *MBM* and type-2 *MBM* lines. Small calli and several shoots were produced at the edge of the explants of the *GUS* line after the explants had been cultured on shoot regeneration medium, for 4 weeks in darkness and then 2 weeks in 16 h light/8 h dark conditions (Fig. 2h). In contrast, innumerable shoots were produced at the explants from type-1 (e.g. *MBM29*) and type-2 (e.g. *MBM8*) lines under the same light and temperature conditions (Fig. 2i, j). On low benzylaminopurine (BA) medium (shoot maintenance medium), under the conditions described above, *GUS* explants only produced a large amount of callus tissue (Fig. 2k), whereas *MBM29* and *MBM8* explants produced both callus and shoots (Fig. 2l, m).

### *MdBBM1* overexpression enhanced cell division of apple leaves

**Figure 3:**
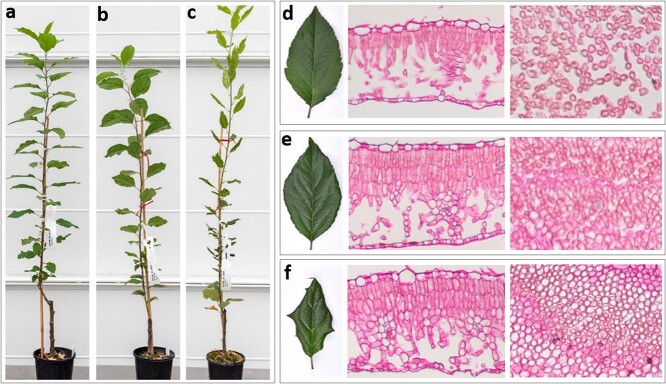
Phenotype analyses of greenhouse-grown apple transgenic lines overexpressing MdBBM1. a–c Plants of a GUS line (a), and two MBM lines carrying 35S-MdBBM1 (b, c) 3 months after being transplanted and grown in a greenhouse. d–f Left panel shows the gross morphology of a fully expanded leaf from a, b and c with the cell density of the leaves shown through cross sections (middle panel) and parallel sections (right panel) through the palisade tissues.

The type-1 *MBM* lines displayed a normal plant architecture in tissue culture. We therefore investigated if these type-1 *MBM* lines showed any phenotypic differences from the *GUS* plants when grown in a greenhouse. After three months of growth in the greenhouse, the type-1 transgenic lines showed two distinct types of plant architecture, named as type-G1 and type-G2. The type-G1 plants were similar to the *GUS* plants (Fig. 3a, b), while the type-G2 plants were more compact and slender (Fig. 3c). The fully expanded leaves of *GUS* plants were smooth, thin, and evenly coloured (Fig. 3d). In comparison, the leaves of type-G1 plants were slightly curly and thicker (Fig. 3e) and the leaves of type-G2 plants were curly, thicker and significantly smaller (Fig. 3f).

To gain insight into the nature of the alteration of leaf development in *MBM* plants that could account for these phenotypic differences, we examined the ultrastructure of the *GUS* and *MBM* apple leaves using light microscopy. Leaf cross-sections of *GUS*, type-G1, and type-G2 *MBM* plants showed that their palisade tissues consist of two, three, and four layers of cells, respectively (Fig. 3d-f). Obviously, the increased palisade cell layers led to the thicker leaves of the *MBM* plants (Fig. 3e, f) compared with those of the *GUS* plants (Fig. 3d). Leaf parallel sections through the palisade tissue showed that the cells were loosely packed with large inter-cellular spaces for the *GUS* plants (Fig. 3d), but densely packed with reduced inter-cellular spaces for the type-G1 and type-G2 *MBM* plants (Fig. 3e, f). This result indicated that overexpression of *MdBBM1* enhanced cell division in apple leaf tissues. It is possible that asymmetry of this cell division and/or cell expansion is the cause of the observed leaf curling.

### *MdBBM1* overexpression effectively enhanced apple transformation

**Figure 4:**
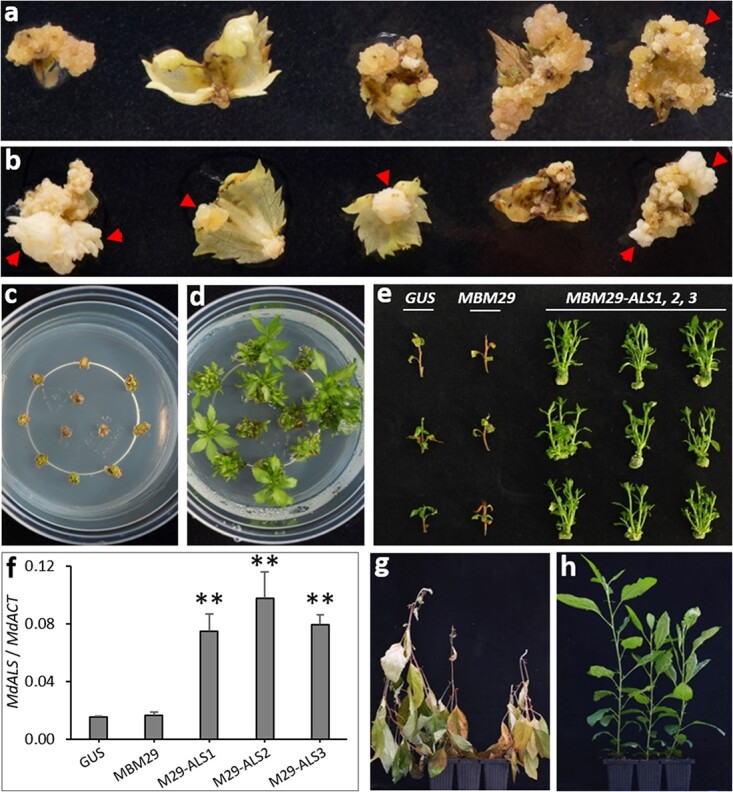
Overexpression of MdBBM1 enhanced apple transformation efficiency. a, b Leaf explants of a GUS line (a) and a 35S-MdBBM1 line (MBM29) (b) after infection with *Agrobacterium tumefaciens* containing the 35S-ALS construct for herbicide resistance and cultured on shoot regeneration medium supplemented with the Glean herbicide (2 μg/L) for three months in dark. Callus tissues and shoot-regenerating foci on these leaf explants are indicated by the red arrows. c, d Shoot-regenerating foci isolated from MBM29 explants without infection or selection (c) or with infection and selection (d) four weeks after transfer to shoot maintenance medium supplemented with 4 μg/L Glean under and a 16-h photoperiod. e Three single shoots of the GUS line, MBM29 and three independent re-transformed lines of MBM29 (MBM29-ALS1, 2, 3) cultured on shoot maintenance medium supplemented with 100 μg/L Glean under a 16-h photoperiod for 4 weeks. f Expression analysis of MdALS in the GUS, MBM29 and three re-transformed MBM29 lines. The values are mean ± SD (n = 3). The asterisks indicate significant differences (P < 0.01) to the GUS line according to a t-test. g, h Plants of the GUS line (g) and MBM29-ALS (h) lines rooted in vitro, established in a greenhouse, then sprayed with 60 mg/L Glean and photographed at 4 weeks post spray.

To select *MBM* lines with minimum alterations in plant morphology and able to be transformed at a higher efficiency than wild-type plants, we carried out re-transformation experiments using a *GUS* line and eight *MBM* lines. The eight *MBM* lines (*MBM10*, *MBM17–18*, *MBM21*, *MBM23–24*, *MBM26*, and *MBM29*) had the type-1 phenotype described earlier when grown in tissue culture. As these lines were kanamycin resistant, a plant transformation vector with *35S-MdALS*, containing a mutant gene encoding acetolactate synthase for conferring resistance to sulfonylurea herbicides, was used in the re-transformation experiments. For each of the *GUS* and *MBM* lines, three independent transformation experiments were performed using 90 to 220 leaf explants per experiment per line (Table 1). At three months post co-cultivation of leaf explants with Agrobacterium tumefaciens harbouring the 35S-MdALS vector, adventitious buds were observed on the leaf explants cultured in darkness on shoot regeneration medium containing 2 μg/L Glean^TM^herbicide (Fig. 4a, b). These adventitious buds and those regenerated from explants without co-cultivation with the *A. tumefaciens* harbouring the 35S-MdALS vector were transferred to the shoot maintenance medium containing a higher concentration of Glean (4 μg/L) and cultured for four weeks in 16 h light/8 h dark. The buds from explants without the co-cultivation did not develop into shoots, and died (Fig. 4c). However, the buds from the re-transformation of *MBM* (e.g. *MBM29*) explants developed into multiple green and healthy-looking shoots (Fig. 4d). These putative transgenic shoots were then transferred to the third selection medium (shoot maintenance medium) containing an extremely high concentration of Glean (100 μg/L). At the same time, control shoots from the *GUS* and *MBM29* line without re-transformation were also transferred to the same high-concentration Glean medium. After 3 weeks, the control shoots from the *GUS* and *MBM29* line were dead and showed no calli formation (Fig. 4e). In contrast, the herbicide-resistant shoots from the re-transformation of *MBM29* explants produced multiple branches and callus tissue at the bottom base of the shoot (Fig. 4e), suggesting that these shoots were re-transformed with the *35S*-*MdALS* construct which conferred strong resistance to the herbicide selection. In addition, expression analysis by ddPCR showed that *MdALS* was overexpressed in three re-transgenic lines (*MBM-29-ALS1*, *2*, and *3*) compared with the *GUS* and *MBM29* lines (Fig. 4f), providing further evidence that these three lines carried the *35S-MdALS* transgene. After rooting on the rooting medium, the *GUS* line and three re-transformed *MBM29-ALS* lines were planted in the greenhouse, and then were sprayed with a high concentration of Glean (60 mg/L). The *GUS* plants died (Fig. 4g), whilst the re-transformed *MBM29-ALS* plants showed normal growth at four weeks after spraying (Fig. 4h), confirming re-transformed plants possessed the *35S-MdALS* transgene.

**Table 1:** Transformation efficiency of apple *GUS* and *MBM* lines

**Lines**	**No. of explants**			**No. of initial shoots**			**No. of transgenic shoots**			**Transformation efficiency (%)**			**Mean transformation efficiency (%)**
	**Exp1**	**Exp2**	**Exp3**	**Exp1**	**Exp2**	**Exp3**	**Exp1**	**Exp2**	**Exp3**	**Exp1**	**Exp2**	**Exp3**	
***GUS***	107	103	189	8	8	15	3	0	4	2.80	0.00	2.12	1.64 ± 1.31 a
***MBM23***	104	170	149	30	41	29	3	7	6	2.88	4.12	4.03	3.68 ± 0.61 ab
***MBM24***	103	106	105	33	35	29	6	5	7	5.83	4.72	6.67	5.74 ± 0.87 b
***MBM26***	109	122	110	25	31	40	6	8	6	5.50	6.56	5.45	5.84 ± 0.56 bc
***MBM18***	113	109	94	31	29	28	8	8	6	7.08	7.34	6.38	6.93 ± 0.44 c
***MBM10***	95	93	97	22	29	25	5	7	8	5.26	7.53	8.25	7.01 ± 1.39 c
***MBM17***	94	90	91	33	33	49	18	16	19	19.15	17.78	20.88	19.27 ± 1.39 d
***MBM21***	91	90	120	68	47	70	23	27	31	25.27	30.00	25.83	27.04 ± 2.31 e
***MBM29***	92	222	220	61	116	109	29	61	56	31.52	27.48	25.45	28.15 ± 2.76 e

Note: Exp1, 2, and 3 represent three independent experiments. Different letters indicate significant differences (*P* < 0.05) by applying Duncan’s multiple comparison test.

The multiple re-transformation experiments showed that the transformation rates of the *MBM* apple lines were much higher than that of the GUS line. The *GUS* line showed a low and inconsistent transformation rate in three re-transformation experiments, from 0% to 2.8% (number of independent transgenic shoots regenerated per 100 leaf explants) (Table 1). The average transformation efficiencies of *MBM10*, *MBM18*, *MBM24*, and *MBM26* were 7.01%, 6.93%, 5.74%, and 5.84%, respectively, which were considerably higher than that of the *GUS* line (1.64%) (Table 1). Most significantly, *MBM17*, *MBM21* and *MBM29* showed consistent and extremely high transformation efficiencies, of 19.27%, 27.04% and 28.15%, respectively (Table 1). In the greenhouse, *MBM10*, *MBM18*, *MBM23–24*, *MBM26*, and *MBM21* were type-G1 plants (with slightly curly leaves), while *MBM17* and *MBM29* were type-G2 plants (with curly leaves). In addition, *MBM23* showed the lowest transformation efficiency (3.68%) among the eight selected *MBM* lines (Table 1), which matched its lowest level of *MdBBM1* expression (Fig. S4). These results showed that overexpression of *MdBBM1* significantly increases the efficiency of apple transformation, and that this enhancement could be linked to the greater capability of the plant to undergo cell division and shoot regenerate shoots.

### *MdBBM1* overexpression changed the expression level of genes involved in plant hormone signal pathways in apple

**Figure 5:**
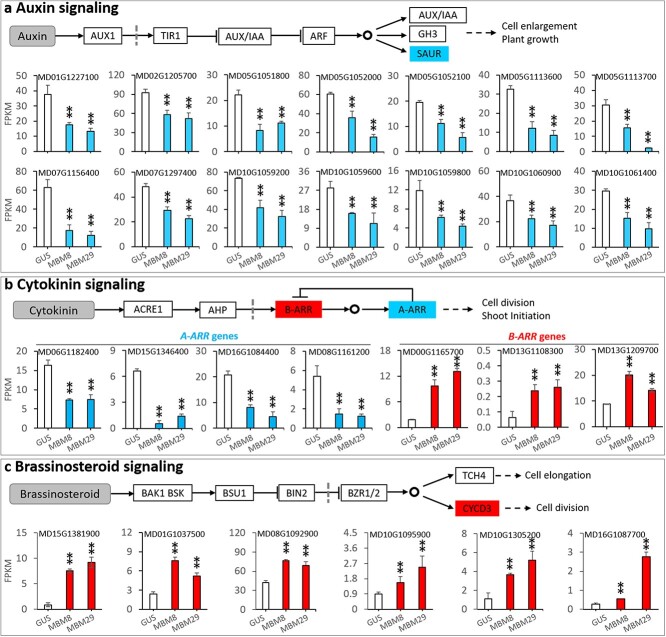
Overexpression of MdBBM1 markedly changed the expression levels of genes in plant hormone signalling pathways. Differentially expressed genes (DEGs) identified by comparison of transcription data of a GUS line and two 35S-MdMBM1 lines (MBM8 and MBM29). Graphical representation of FKPM values from RNA-seq data of various classes of genes are shown, as are the identities of these genes (MD numbers) in the apple reference genome. a Small auxin up-regulated RNA (SAUR) genes of the auxin signalling pathway leading to cell enlargement and plant growth (upper panel). Fourteen SAUR genes significantly down-regulated in the MBM8 and MBM29 lines compared with the GUS line are shown. b Type-A ARR (A-ARR) and type-B ARR (B-ARR) genes encoding suppressors and activators, respectively, in the cytokinin signalling pathway leading to cell division and shoot regeneration (upper panel). Four A-ARR and three B-ARR genes significantly down-regulated and up-regulated, respectively, in MBM8 and MBM29 lines compared with the GUS line are shown. c D-class cyclin (CYCD) responds to brassinosteroid signalling for promoting cell division (upper panel). Six CYCD genes up-regulated in MBM8 and MBM29 lines compared with the GUS line are shown. FPKM values are means ± SD of three RNA-seq libraries. Red bars represent up-regulation of genes and blue bars represent down-regulation of genes in the MBM8 and MBM29 lines compared with the GUS line. The values in a, b and c are mean ± SD (n = 3). The asterisks in a, b and c indicate significant differences (P < 0.01) to the GUS line according to a t-test.

To understand the molecular basis of the enhanced shoot regeneration and transformation by overexpressing *MdBBM1* in apple, we analysed genome-wide gene expression changes using RNA sequencing data. By comparing the gene expression profiles in young leaves between the *GUS* line and two *MBM* lines (*MBM8* and *MBM29)*, we identified dramatic differences in global gene expression patterns. A total of 1770 differentially expressed genes (DEGs) (padj <0.01 and fold change >1.5) were identified between the *GUS* and *MBM8* line, consisting of 673 up-regulated genes and 1097 down-regulated genes in *MBM8* (Fig. S5a). A total of 5635 DEGs were identified between the *GUS* and *MBM29* line, consisting of 2646 up-regulated genes and 2989 down-regulated genes in *MBM29* (Fig. S5a). Around 66% of genes up-regulated (447 of 673 genes) and 75% of genes down-regulated (825 of 1097 genes) in *MBM8* were also up- and down-regulated, respectively, in *MBM29* (Fig. S5b, c), indicating that overexpression of *MdBBM1* regulated a similar set of genes in two different transgenic lines. The 1770 DEGs in *MBM8* and 5635 DEGs in *MBM29* were then annotated with KEGG terms (Fig. S6a, b), and revealed changes in expression of a number of key genes in hormone pathways related to cell division and shoot regeneration (Fig. 5).

*Auxin/indole-3-acetic acid* (*Aux/IAA*) [21], *Gretchen Hagen 3* (*GH3*) [26], and *small auxin up-regulated RNA* (*SAUR*) [27] are the early auxin response genes that play important roles in plant auxin signal transduction. Transcript levels of all the *Aux/IAA* and *GH3* genes were unchanged in *MBM8* or *MBM29* compared with the *GUS* line (data not shown). However, *SAUR* genes were enriched in the DEGs between the *GUS* line and two *MBM* lines (Fig. 5a). We identified 80 *SAUR* genes from the “Golden Delicious” GDDH13 apple reference genome. Of these *SAUR* genes, 14 showed significant reduction of transcript level in *MBM8* and *MBM29* compared with the *GUS* line, 22 showed slight reduction of transcript levels in both *MBM8* and *MBM29*, 44 showed no difference between the two *MBM* lines and the *GUS* line (Table S2). The down-regulated *SAUR* genes mostly located on chr5 and chr10 as tandem repeats (Fig. S7a, b). The 14 significantly down-regulated genes (Fig. 5a, Fig. S7a) were analysed together with 79 Arabidopsis *SAUR* genes to determine their phylogenetic relationship (Fig. S7c). The analysis showed that protein sequences of MD10G1061400 clustered together with Arabidopsis AtSAUR41, while MD01G1227100, MD02G1205700, MD07G1156400, and MD07G1297400 showed a closer relationship to Arabidopsis AtSAUR55 and AtSAUR63 (Fig. S7c). The other nine apple SAUR sequences clustered into a group with Arabidopsis AtSAUR2 and AtSAUR17 (Fig. S7c).

Type-A Arabidopsis Response Regulators (A-ARRs) and Type-B ARRs (B-ARRs) are negative and positive regulators of cytokinin responses, respectively. Their activities are linked to cell division and shoot initiation [12, 28, 29]. The transcriptome analysis showed that *A-ARR* and *B-ARR* genes were enriched in the DEGs between the *GUS* line and two *MBM* lines (Fig. 5b). Of the ten *A-ARR* genes identified in the apple reference genome, four genes (MD06G1182400, MD08G1161200, MD15G1346400, and MD16G1084400) were significantly down-regulated in both *MBM* lines (Fig. 5b, Fig. S8a). In contrast to *A-ARR* genes, eight *B-ARR* genes were up-regulated in the *MBM* lines (Fig. S8b). Of these genes, MD00G1165700, MD13G1108300, and MD13G1209700 were significantly up-regulated in both *MBM* lines, while the other five genes were significantly up-regulated DEGs in only one of the two *MBM* lines (Fig. 5b; Fig. S8b). The phylogenetic analysis showed that apple A-ARR Md06G1182400 and MD16G1084400 clustered together with Arabidopsis ARR5/6 and ARR3/4, respectively (Fig. S8c). MD08G1161200 and MD15G1346400 showed a closer relationship with Arabidopsis ARR16/17 (Fig. S8c). Apple B-ARR MD00G1165700 and MD13G1108300 clustered with Arabidopsis ARR11/14, and MD13G1209700 with ARR19 (Fig. S8c).

The D-class cyclins (CYCDs), particularly CYCD3, play a key role in cytokinin or brassinosteroid responses and positively regulate cell division and plant regeneration [11, 30–33]. The expression levels of most cyclin genes, including *CYCA*, *CYCB, CYCP* and *CYCT*, did not differ between the *MBM* and *GUS* lines (Fig. S9a). However, six *CYCD* genes were significantly up-regulated in the two *MBM* lines compared with the *GUS* line (Fig. 5c, Fig. S9b). The six were classified as CYCD1 (MD16G1087700), CYCD3 (MD08G1092900 and MD10G1095900), CYCD4 (MD01G1037500 and MD15G1381900) and CYCD6 (MD10G1305200) using a phylogenetic analysis together with annotated CYCDs of Arabidopsis (Fig. S9b).

## Discussion

### Improving apple transformation using BBM overexpression

A highly efficient transformation system is essential for the timely production of a sufficient number of transgenic plants required for reliable and meaningful functional genomics studies, and for the identification of transgenic lines with the optimal phenotypes. Although such systems are available for model plant species and some annual crop species [34, 35], they are still not available for the majority of woody perennial species [36]. Most woody plant transformation systems previously reported have a sub-optimal efficiency [2–4, 37], and although they have played critical roles in revealing gene function in tree species on a small scale [37–40], they are not effective for analysing the function of a large number of genes in trees in a timely manner. Although many candidate genes can be identified rapidly using modern genomics technologies, the limitations of most tree transformation systems remains a severe impediment to determining their function in tree species. The science community would greatly benefit from having access to more reliable tree transformation technologies in the genomics era.

In this study, we demonstrated that apple transformation efficiency can be dramatically increased by using BBM, a potent regulator of plant embryogenesis and organogenesis. The production of apple transgenic plants expressed as the number of transgenic plants produced per 100 leaf explants was increased from 0–3% to 20–30% (Table 1). Large-scale improvement of this magnitude has been shown in herbaceous species [13, 20], but not in woody fruit tree species. Our success with a fruit tree species may be explained by the differences between the approach we used here and those used previously.

There are two major differences between the approach we describe here and those reported previously, which have shown no significant enhancement of transformation in tree species. Firstly, previous studies included the *BBM* gene construct in the same vector as the candidate genes and tested for enhanced transformation efficiency in the primary transformation experiments [19]. In contrast, we produced numerous stable transgenic plant lines with the *BBM* transgene, then selected the lines with improved rates of cell division, regeneration and transformation, and used these lines as the starting materials for subsequent re-transformation using a vector containing a different selectable marker and a gene of interest or candidate gene. The transformation efficiency was determined in the re-transformation experiments rather than in the primary transformation experiments. In the re-transformation experiments, all cells of the explants should contain the *BBM* transgene and thus had the same potential for a high rate of cell division. In contrast, in the primary transformation experiment, only a very low proportion of the explant cells may have taken up the *BBM* transgene, and thus cells may have variable potential for enhanced cell division. So re-transformation should have a greater potential than the previous single transformation approach for significant and consistent improvement of the transformation rate.

The second difference is the constitutive versus inducible activity of BBM. In previous approaches, undesirable changes of phenotype due to BBM activity were avoided by using a BBM-GR fusion protein. The presence of the GR element confines the fusion protein to the cytoplasm, preventing BBM activity. Enhanced transformation and regeneration in tissue culture was achieved by release of the sequestration, through the addition of the DEX inducer, allowing the BBM-GR to enter the nucleus and thus enabling subsequent BBM activity. The absence of the inducer after the transformation and regeneration stages prevents BBM activity having undesirable effects on the plant phenotypes [16, 19]. Although we found that the DEX-inducible BBM-GR approach worked well in tobacco, demonstrating our transformation constructs worked as expected, this was not the case in apple. Our findings in apple showed shoot regeneration was enhanced in the absence of DEX and the addition of DEX saw no further enhancement, suggesting that the BBM-GR protein was not completely sequestered to the cytoplasm in the absence of DEX, and that at least some of the fusion protein could enter the nucleus. To overcome this problem, we used constitutively active BBM and identified and selected transgenic lines with a balanced rate of BBM activity that enhanced regeneration and transformation but did not cause too much in the way of detrimental phenotypes. The success of our approach means that it may be applied to other tree species recalcitrant to transformation and regeneration.

### Identifying BBM transgenic plants with an enhanced transformation efficiency but without aberrant phenotypes

Although over-expression of *BBM* can enhance transformation and plant regeneration, the transgenic plants may show aberrant phenotypes that affect their use in further studies. In our study, some *MdBBM1* over-expression lines of apple were compact, short and with more branching (type-2) in the tissue culture (Fig. 2c), similar to the phenotype described in previous reports [14, 17, 21]. The compact plants produced small leaves not ideal for use in re-transformation experiments. Fortunately, we identified other *MdBBM1* over-expression lines that displayed a more normal phenotype (type-1) that was almost indistinguishable from WT apple plants in tissue culture. These lines provided healthy leaf tissue ideal for further transformation experiments. When grown in the greenhouse, some type-1 plants showed compact plant architecture with curling leaves (type-G2) (Fig. 3c), whereas other type-1 plants showed a normal architecture and leaf morphology (type-G1), identical to plants with a *GUS* transgene. Furthermore, these type-G1 lines, especially *MBM21*, showed a significantly higher transformation efficiency than the *GUS* line. Therefore we have identified *MBM* lines that can be used for highly efficient apple transformation but show no aberrant phenotypes. These lines will be extremely useful for future studies analysing gene function in apple and will significantly reduce the labour and consumable costs to produce appropriate numbers of transgenic plants for such studies. A similar result with no obvious aberrant phenotype was reported for the transgenic maize plants overexpressing *ZmBBM2*^20^. Therefore, selection of transgenic lines with a level of BBM balanced to provide an increase in transformation efficiency without deleterious plant phenotypes is probably applicable to other plant species, particularly those species without a reliable inducible system to regulate gene expression or function of *BBM* or similar genes.

### Overexpression of BBM altering hormone response

The genetic basis for differences in plant regeneration is still poorly understood. However, an appropriate ratio between the plant hormones auxin and cytokinin is known to determine the developmental fate of plant cells in tissue culture, e.g. whether the cells do or do not differentiate into shoots. Generally, a high ratio of cytokinin to auxin stimulates shoot regeneration [41, 42]. In this study, the leaf explants from apple *MBM* transgenic lines tended to regenerate more shoots even on medium with a low amount of cytokinin (1 mg/L BA) (Fig. 2l, m), suggesting that cytokinin response was stimulated in the *MBM* plants.

In the cytokinin signalling pathway, ARRs are the primary regulators linking cytokinin signals to plant growth and development. In Arabidopsis, loss of function mutations in B-ARRs, which act as positive regulators of primary cytokinin response genes, reduces shoot regeneration [12, 43], and over-expression of A-ARRs, especially *AtARR3*, *AtARR5–6* and *AtARR16–17*, which are negative regulators of cytokinin signalling, also reduces shoot regeneration [29]. Our transcriptome analysis showed that four *A-ARR* genes were down-regulated and three *B-ARR* genes were up-regulated in the *MBM* plants (Fig. S8a, b). Interestingly, the four down-regulated *A-ARRs* cluster together with *AtARR3/4*, *AtARR5/6*, and *AtARR16/17*, respectively, in phylogenetic analysis (Fig. S8c). These results are consistent with the negative role of A-ARRs and positive role of B-ARR in the regulation of cytokinin signalling, and this further suggests that overexpression of BBM enhances the cell’s response to cytokinin, thus improving shoot regeneration of the transgenic *MBM* plants.

In addition, *SAUR* genes, important for plant responses to auxins [27], were down-regulated in *MBM* plants (Fig. S7a) and may reduce leaf tissue sensitivity to auxins. Plant development requires cell division, expansion and differentiation with cell division occurring primarily in meristems and primordia. Of the 14 significantly down-regulated *SAURs* in the apple *MBM* lines, five clustered together with Arabidopsis *AtSAUR41* and *AtSAUR63* genes (Fig. S8c), which play key roles in promoting cell expansion and auxin transport [44, 45]. It is not unreasonable to suggest that down-regulation of these five apple *SAUR* genes may alter the balance between cell division and expansion and thus promote adventitious shoot meristem formation. The pattern of enhanced cytokinin-response and supressed auxin-response in *MBM* transgenic plants is also consistent with our general observation that shoot regeneration requires more concentration of cytokinin compared to auxin in the media.

CYCDs play an important role in cytokinin- or brassinosteroid-response to promote cell division [11]. Previous reports have shown that over-expression of CYCD3 could effectively induce the shoot regeneration [11, 31]. Interestingly, in the *MBM* plants the transcriptome analysis revealed six *CYCD* genes, including two CYCD3 genes, were up-regulated (Fig. S9a), suggesting that *CYCDs* were downstream genes of *BBM* in enhancing apple shoot regeneration. Taken together, our transcriptome analyses indicates that MdBBM1 could effectively enhance apple shoot regeneration through regulating the crosstalk between auxin, cytokinin and brassinosteroid signals.

In conclusion, overexpression of *MdBBM1* can significantly improve the efficiency of apple transformation and regeneration, and produce healthy transgenic plants for re-transformation studies. It is evident that the improvement is linked to MdBBM-mediated regulation of the cross-talk between the signalling pathways of auxin, cytokinin and brassinosteroid through modelling the expression levels of *SAUR*, *A/B-ARR* and *CYCD* genes. The approach we have described here for use in apple should be applicable in other plant species recalcitrant to transformation, and thereby help to overcome some of the limitations of studying the molecular genetics of these species, which are often of economic significance.

## Materials and methods

### Tissue culture media and culture conditions

Five types of apple tissue media were used in this study. These included the four media (shoot maintenance medium, shoot regeneration medium, rooting medium and MS20 medium) described by Yao et al. [4], and a leaf expansion medium (Table S3). All media were adjusted to pH 5.8 with NaOH and then autoclaved at 1.1 kg cm^−2^ (121°C) for 15 min. For transformation experiments the antibiotics: cefotaxime (Claforan, Roussel NZ Ltd); kanamycin sulphate (Sigma); and the herbicide Glean (Du Pont, active agent chlorsulfuron) were filter-sterilized and added where appropriate to media after autoclaving. For maintaining shoot cultures and rooting, 290-mL plastic containers (Aimed TC290SP, 8 × 6 cm, diameter × height) each containing 50 mL medium were used to culture 8–10 shoots per container. For co-cultivation and shoot regeneration experiments, Petri dishes (8.5 cm diameter) each containing 20 mL medium were used to culture up to 15 explants per plate. The conditions of the plant tissue culture room were set at 24°C and 16-h photoperiod (30 μmol m^−2^ s^−1^).

### Vector construction

The plant transformation vectors pART27–10 containing the CaMV*35S-GUS* construct and pAAM2 overexpressing a mutant of *MdALS* gene were constructed in previous studies [46]. To regulate the localization of BBM in cell compartments, a DNA fragment was designed to fuse the apple *MdBBM1* with the rat glucocorticoid receptor (GR) ligand binding domain (LBD) (amino acids 512–795) [16], with added restriction enzyme sites (*Bam*HI, *Eco*RI, and *Xba*I) to facilitate cloning (Fig. S10). The CDS sequence of *MdBBM1* is based on the gene model of sequences of MDP0000871080 [24] with the un-predicted small exon sequence (GTTTATCTG) included. The designed DNA was synthesized and then cloned into the *Bam*HI and *Xba*I sites of the plant transformation vector pSAK778 [47], between the CaMV35S promoter and *ocs* terminator, to generate the vector pSAK778-MdBB1-GR (Fig. S11A). The construct was then digested by *Eco*RI to remove the GR domain, and then self-ligated to generate the construct pSAK778-MdBBM1 (Fig. S11B). All three constructs were sequenced and confirmed to be correct.

### Plant transformation

Tobacco (*N. tabacum* “Samsun”) transformation was carried out as previously described [4]. Following co-cultivation with *A. tumefaciens* LBA4404 containing a construct of interest, regeneration of transgenic shoots was selected using 100 mg/L kanamycin sulphate.

Apple (*M. domestica* “Royal Gala”) transformation methods were modified from previously described protocols [3, 4]. To provide leaf tissues suitable for transformation, wild-type “Royal Gala” apple shoots were cultured on shoot maintenance medium by sub-culturing at four-week intervals. These shoots were transferred to the leaf expansion medium and cultured for four weeks before young, expanding leaves were harvested for transformation experiments. Each leaf was cut transversely into three leaf explants that were infected with a suspension of *A. tumefaciens* LBA4404 cells containing a construct of interest (pART27–10, pSAK778-MdBBM1-GR, or pSAK778-MdBBM1). The infected leaf explants were co-cultivated on the shoot regeneration medium for three days in the dark at 22°C. Following the co-cultivation, the explants were transferred onto the shoot regeneration medium supplemented with 200 mg/L cefotaxime and 100 mg/L kanamycin sulphate (15 explants per plate), incubated in the dark at 22°C for four weeks. After three months in the dark with two transfers to fresh medium, kanamycin-resistant shoots were regenerated and were transferred to the shoot maintenance medium supplemented with 200 mg/L cefotaxime and 100 mg/L kanamycin sulphate for shoot multiplication and elongation. The shoots were then micro-grafted to “M9” rootstock in a greenhouse for phenotyping.

For re-transformation, leaf explants of a *GUS* line and eight *MBM* lines were co-cultivated with A. tumefaciens LBA4404 containing a construct of interest (the pAAM2). For pAAM2 construct, shoot regeneration was under herbicide selection using 2 μg/L Glean in the dark at 22°C for three months. The regenerated shoots were then transferred to shoot maintenance medium supplemented with 4 μg/L Glean and incubated in a plant tissue culture room with 16-h photoperiod for one month, followed by transfer to medium supplemented with 100 μg/L Glean for another month and then transferred to rooting medium containing 100 μg/L Glean. The rooted plants were transferred to the greenhouse and then sprayed with 60 mg/L Glean as previously described [3] to finally confirm herbicide resistance.

### Analysis of the *MdBBM1* transcript level in transgenic tobacco and apple plants

Leaves were collected from different transgenic lines grown in tissue culture, immediately frozen in liquid nitrogen and stored at −80°C. From these leaf tissues, total RNA was extracted using a Spectrum™ Plant Total RNA Kit (Sigma-Aldrich, USA) following the manufacturer’s instructions. The residual DNA in the RNA samples was removed by digestion using a TURBO DNA-free Kit™ (Ambion, USA). The first- and second-strand complementary DNA was synthesized using a cDNA Synthesis Kit (TaKaRa, Japan). After cDNA synthesis from these RNAs, the transcript levels of *MdBBM1* and references genes (*Actin* and *PP2A)* were analysed using droplet digital PCR (ddPCR) with primers listed in Table S4. The ddPCR was performed using EvaGreen SuperMix (Bio-Rad, USA) Reagents and QX 200™ Droplet Digital PCR System (Bio-Rad, USA).

### Histological analysis and microscopy

The fully expanded leaves were collected from three-month-old plants of the *GUS* line and two *MBM* lines grown in the greenhouse. Leaf segments (0.5 cm × 0.5 cm) across the main vein were collected and fixed in FFA (4% formalin, 50% alcohol and 5% acetic acid in water to 100%). After fixation, the tissues were dehydrated, embedded in paraffin wax and sectioned as previously described [40]. The leaf sections were stained with ruthenium red and photographed using an Olympus Vanox AHT3 microscope.

### RNA sequencing and transcriptome analysis

To explore the molecular basis underlying enhanced shoot regeneration in *MdBBM1* overexpression lines, differences in gene expression between the *GUS* line and two *MBM* lines (*MBM8* and *MBM29*) were analysed using RNA-seq data. To construct RNA-seq libraries, young leaves were collected from three-month-old plants of the three lines grown in a greenhouse. Three biological replicates were collected for each line. From these leaf samples, total RNA was extracted using a Spectrum™ Plant Total RNA Kit (Sigma-Aldrich, USA) following the manufacturer’s instructions. The RNA quantity and quality were assessed by a NanoDrop ND-1000 Spectrophotometer (Thermo Scientific, USA) and an Agilent 2100 Bioanalyzer (Agilent Technologies, California, USA). From the total RNA sample, poly (A) mRNAs isolation was performed using oligo-dT attached to magnetic beads. The purified mRNAs were fragmented using super sonication and then subjected to first- and second-strand cDNA synthesis using random hexamer primers. The cDNA libraries were prepared using the NEBNext®Ultra™ RNA Library Prep Kit (New England Biolabs, USA) following the manufacturer’s instructions, and sequenced from both ends of the cDNA using an Illumina HiSeq™ 2000 sequencer at Novogene (Beijing, China).

The RNA-seq reads were cleaned using trimmomatic 0.38 and then mapped to the apple GDDH13 reference genome^23^ using Hisat v2.0.4 tools. The number of clean reads mapped to each gene was counted using the software HTSeq v0.5.4. Gene expression levels were calculated based on Fragments Per Kilobase of transcript per Million mapped reads (FPKM). Differential expression analysis were performed using the DESeq R package. The *p-*values were adjusted using the Benjamini and Hochberg’s approach for controlling the false discovery rate. Genes with padj <0.01 and fold change >1.5 were considered as DEGs. DEGs were subsequently analysed using Volcano plot, Venn diagram, and KEGG pathway enrichment analysis. Venn diagrams and Volcano plots were made using the TBtools v1.092 software [48]. KOBAS software was used to test the statistical enrichment of differential expression genes in KEGG pathways.

## Supplementary Material

Web_Material_uhab014

## Data Availability

The data used to support the findings of this study are included within the article.
