# Supplementary material for: Significant improvement of apple (Malus domestica Borkh.) transgenic plant production by pre-transformation with a Baby boom transcription factor
Source: Hortic Res. 2022 Jan 28;9:uhab014. doi: 10.1093/hr/uhab014 (PMC8795818; doi:10.1093/hr/uhab014)
Supplement: Web_Material_uhab014 [file web_material_uhab014.zip › Supplemental Figures.docx]

Supplementary figures for

**Significant improvement of apple (*Malus domestica*) transgenic plant production by using Baby Boom transcription factor**

Jiajing Chen^1,2^, Sumathi Tomes^1^, Andrew P. Gleave^1^, Wendy Hall^1^, Zhiwei Luo^1^, Juan Xu^2^, Jia-Long Yao^1,3 *^.

^1^ The New Zealand Institute for Plant and Food Research Limited, Private Bag 92169, Auckland 1142, New Zealand

^2^ Key Laboratory of Horticultural Plant Biology (Ministry of Education), College of Horticulture and Forestry, Huazhong Agricultural University, Wuhan 430070, China

^3^ Zhengzhou Fruit Research Institute, Chinese Academy of Agricultural Sciences, Zhengzhou 450009, China

* Corresponding authors:

Jia-Long Yao, e-mail: Jia-Long.Yao@plantandfood.co.nz


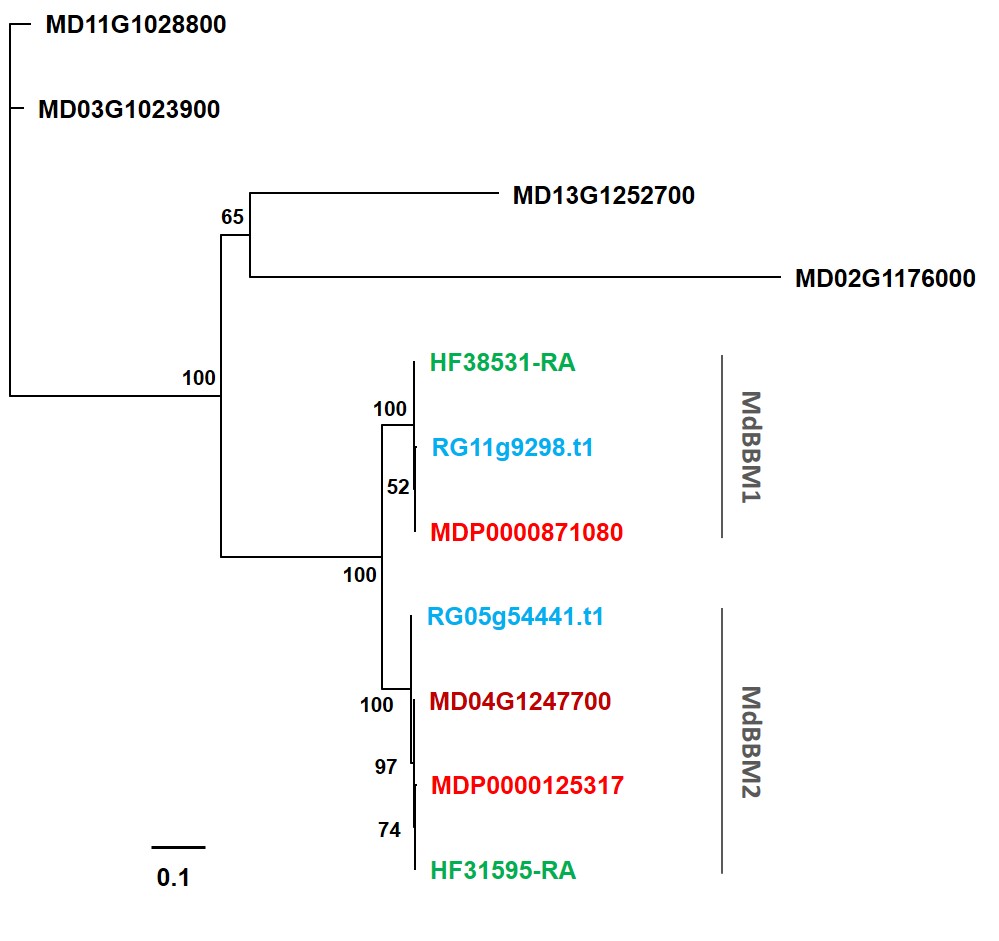


**Supplemental Figure 1. Phylogenetic tree of BBM and closely related proteins identified from four apple reference genomes.**

A neighbor-joining tree was constructed using MEGA 7.0 with 1000 bootstrap replicates after the protein sequences of apple MdBBMs and the related proteins were aligned by ClustalX 2.1.


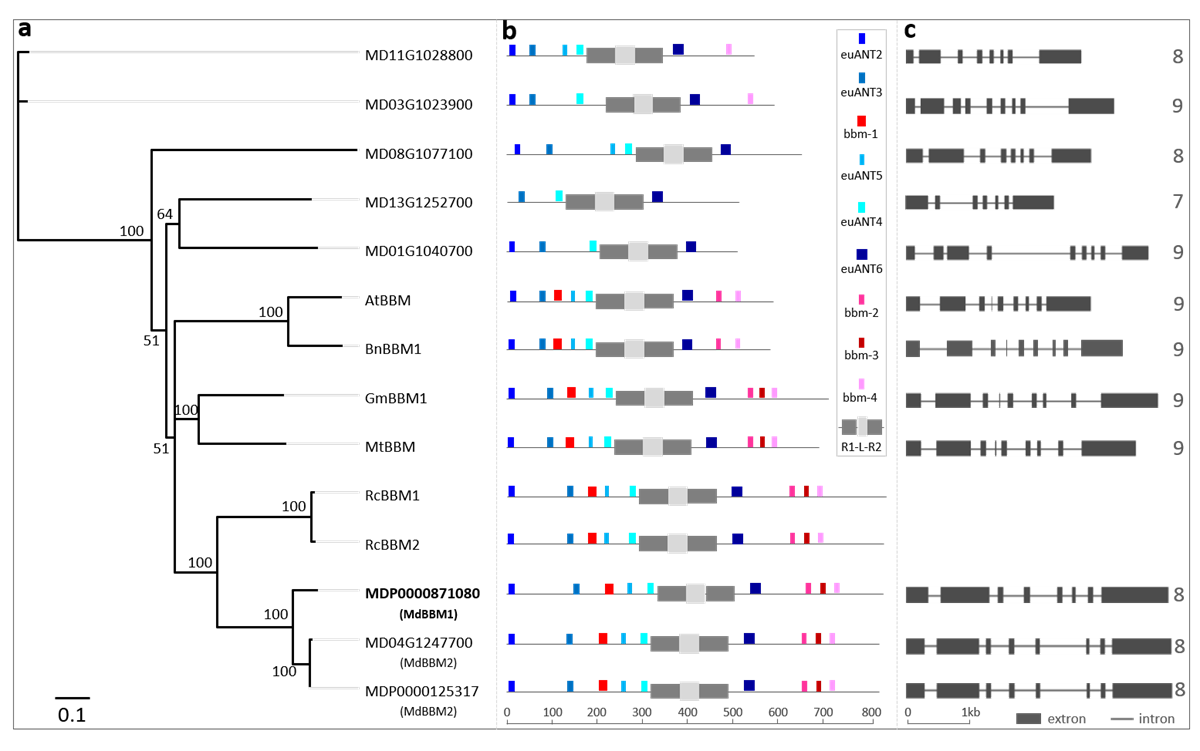


**Supplemental Figure 2. Comparison of *BBM* genes of in apple and other plants.** **a** A neighbor-joining tree was constructed using MEGA 7.0 with 1000 bootstrap replicates after the protein sequences of apple MdBBMs and related proteins, and BBMs with function identified from other plant species were aligned by ClustalX 2.1. **b** Conserved motifs, including euANT2, 3, 4, 5, 6; bbm1, 2, 3, 4, and R1-L-R2, were identified from the protein sequences used in (a) using MEME Suite analysis tool. The ‘R1-L-R2’ represents two AP2 domain repeats and a link sequencing between them. **c** Exons and introns were predicted for the genes encoding the protein sequences in (a), except for *RcBBM1* and *RcBBM2,* for which complete genomic DNA sequences were not reported.


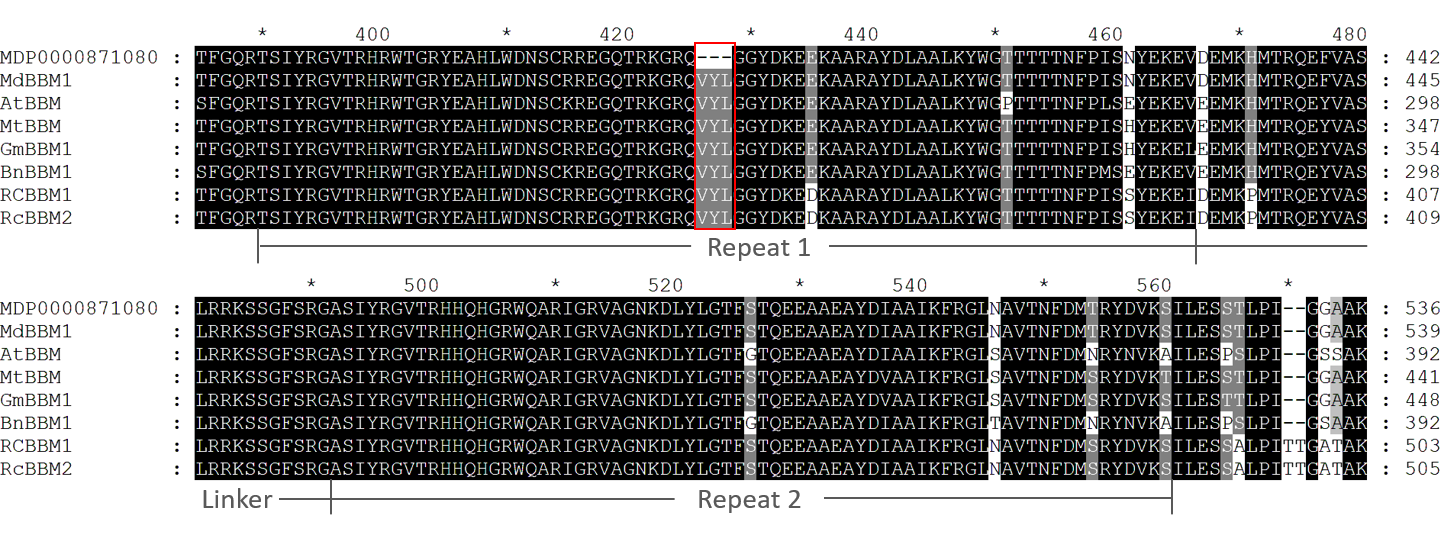


**Supplemental Figure 3. Alignment of the AP2 domain amino acid sequences of MdBBM1 and BBM proteins from other plant species.** MDP0000871080 represents the gene model sequence of MdBBM1 in the ‘Golden Delicious’ apple reference genome; MdBBM1represents the correct sequence by adding the three amino acids ‘VYL’ to MDP0000871080.

**
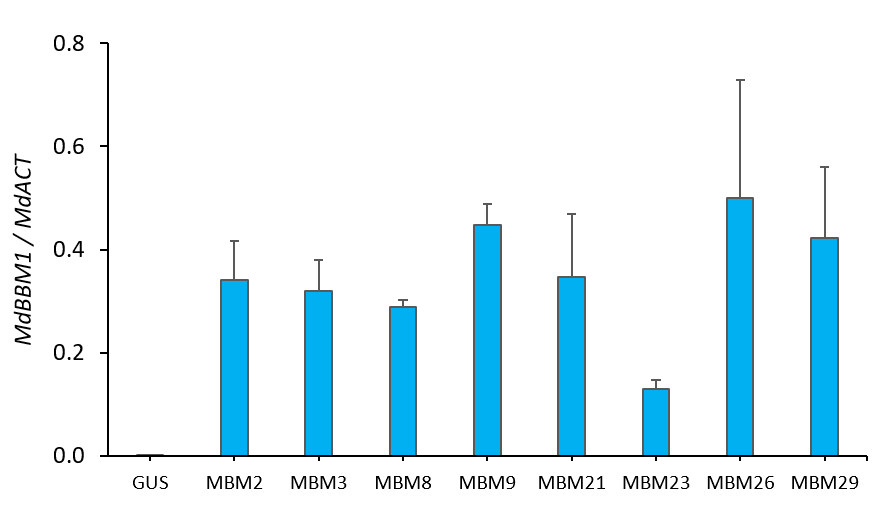
**

**Supplemental Figure 4. Overexpression of *MdBBM1* mRNA was detected in eight apple lines transformed with *35S-MdBBM1*.** The ratio of mRNA level between *MdBBM1* and *MdACT* in leaves of a *GUS* line and eight *MdBBM1* (*MBM*) lines was determined using dd-PCR. The values are means ± SD (n=3).

**
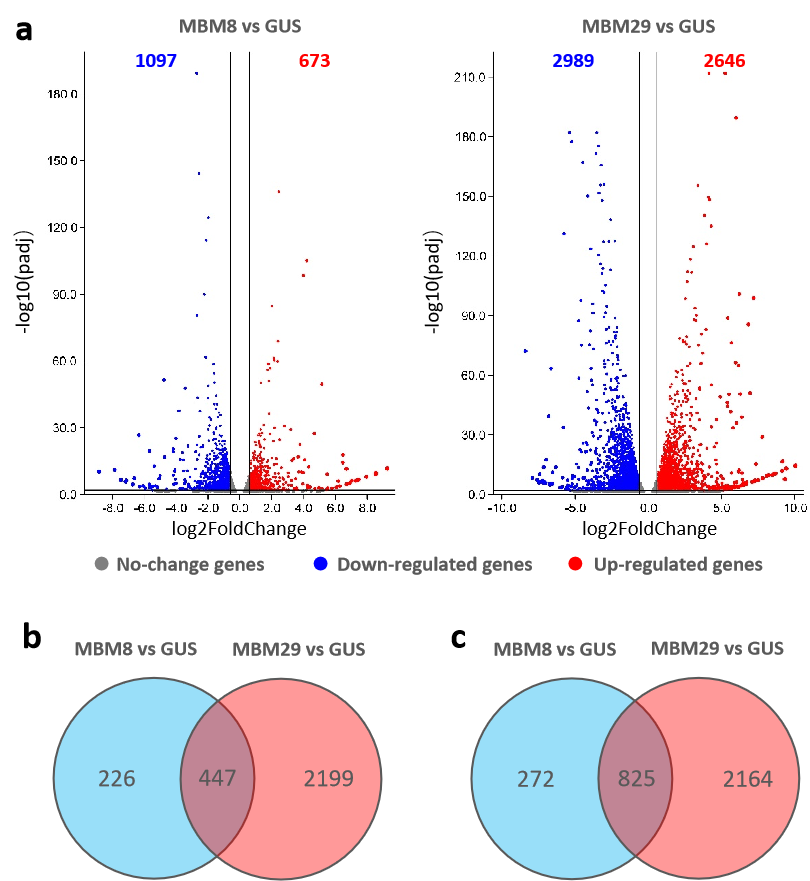
**

**Supplemental Figure 5. DEGs identified by transcriptome analysis by comparisons of *MBM8* and *MBM29* to *GUS* plants. a** Volcano diagrams of DEGs showing up- and down-regulated genes when *MBM* line is compared with the *GUS* line. Number of up-regulated (red) and down-regulated (blue) genes are shown; **b, c** Venn plot of up-regulated (**b**) and down-regulated (**c**) DEGs in *MBM8* and *MBM29* compared with the *GUS* line.

**
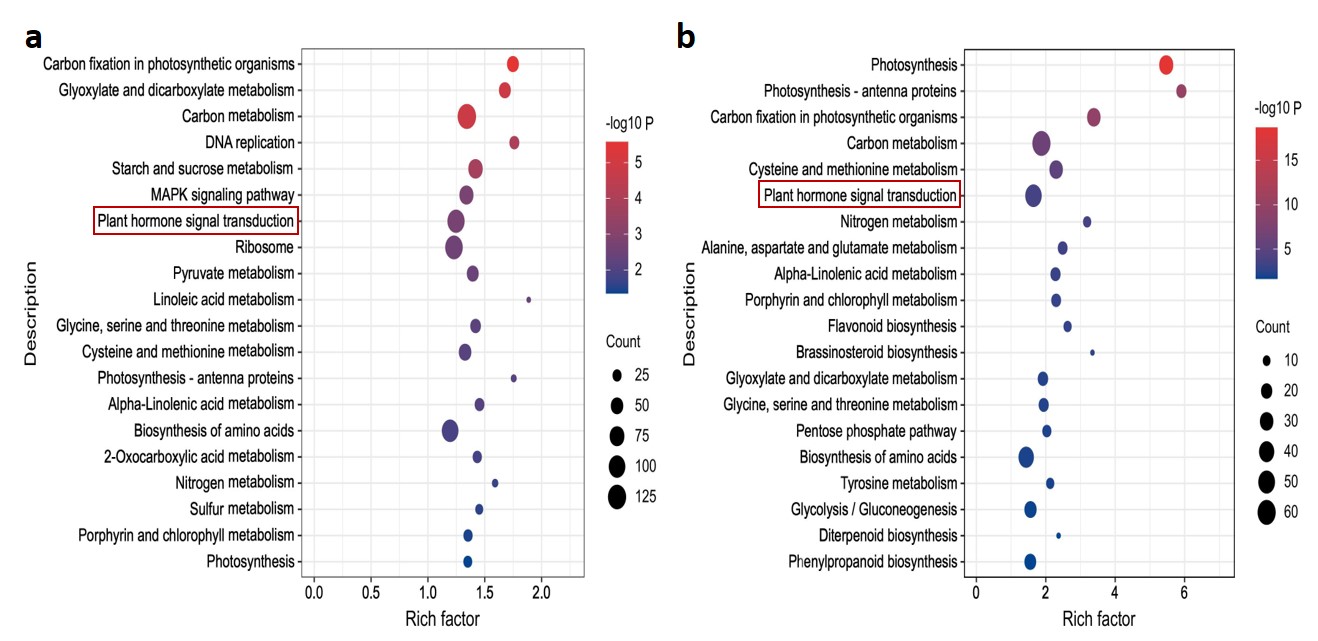
**

**Supplemental Figure 6. KEGG analysis of DEGs.** Pathways with enrichment of DEGs were identified based on comparisons of transcriptome data between *MBM8* and *GUS* plants (a), and between *MBM29* and *GUS* plants (b).

**
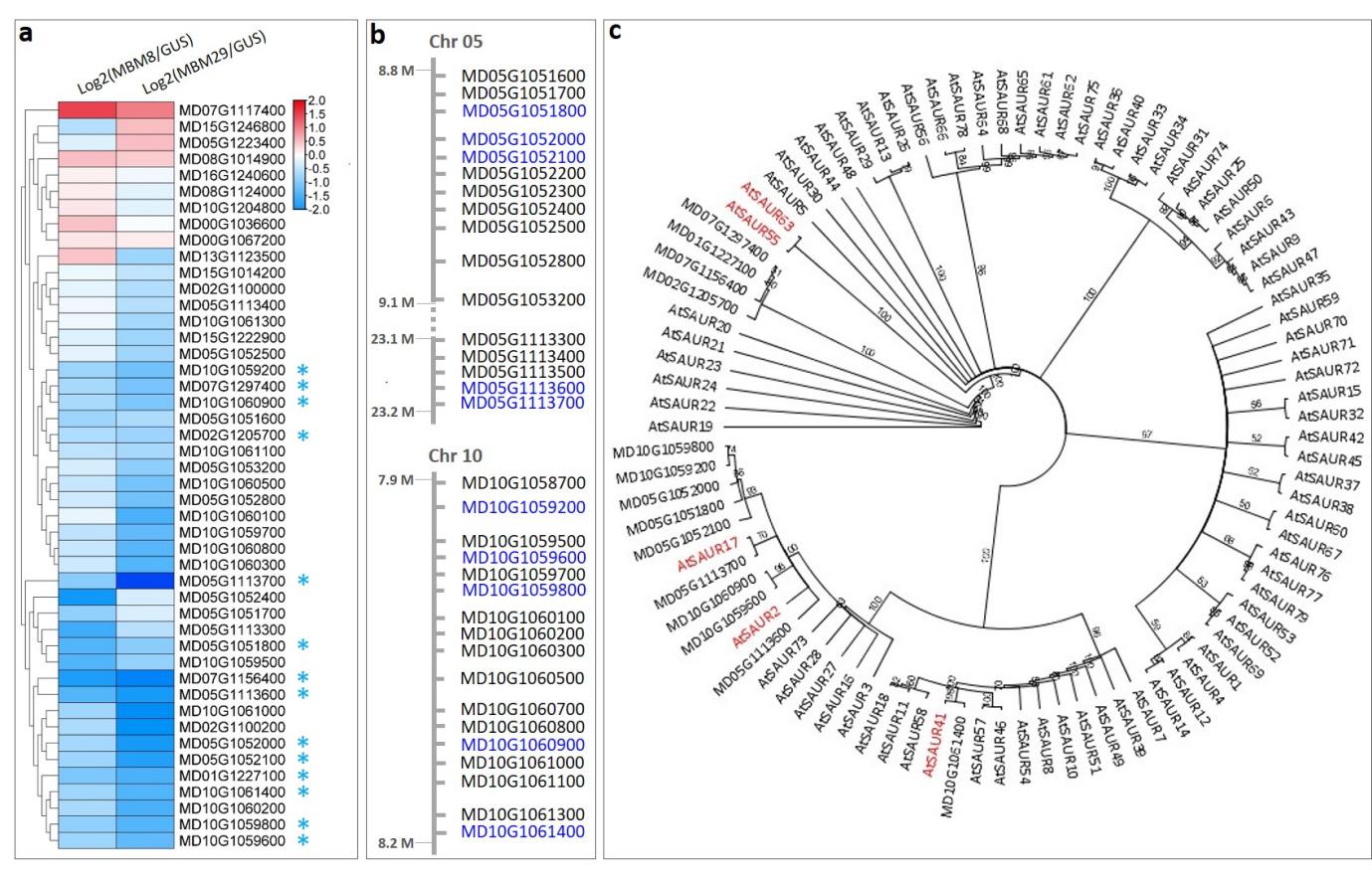
**

**Supplemental Figure 7. Analyses of Small Auxin Up-regulated RNA (*SAUR*) genes. a** Heat map of *SAUR* gene transcript levels in *MBM8* and *MBM29* transgenic plants compared with *GUS* plants. The genes marked by the blue asterisk were down-regulated in both *MBM8* and *MBM29* plants compared with the *GUS* plants. The 34 *SAUR* genes with low expression levels (Table S2) were eliminated before conducting the heat map analysis. The colour scale represents log_2_fold change of FPKM values. **b** *SAUR* genes were found to be tandemly located on chr5 and chr10. The genes marked by the blue colour were down-regulated in both *MBM8* and *MBM29* plants compared with the *GUS* plants. **c** Phylogenetic tree of protein sequences of 14 DEGs from (a) and 79 Arabidopsis SAUR proteins. The *AtSAURs* marked by the red colour were clustered together with the down-regulated *MdSAURs* in two *MBM* lines.

**
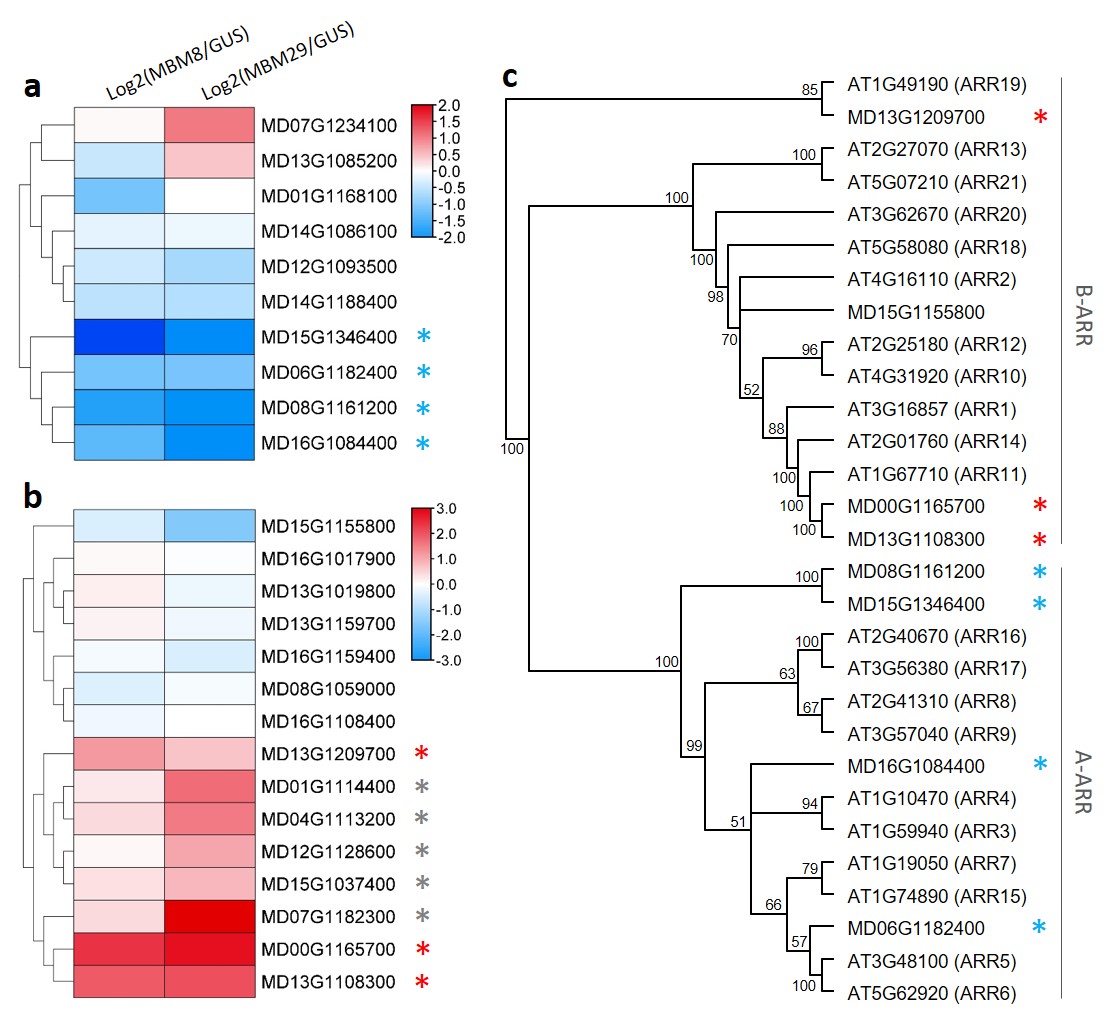
**

**Supplemental Figure 8. Expression changes of *A-ARR* and *B-ARR* genes between *MBM* and *GUS* plants. a** Expression changes of apple *A-ARR* genes between *MBM* and *GUS* plants. The blue asterisks indicate genes down-regulated in both *MBM8* and *MBM29* compared with *GUS* plants. **b** Expression changes of apple *B-ARR* genes between *MBM* and *GUS* plants. The red asterisks indicate genes up-regulated in both *MBM8* and *MBM29*, and the grey asterisks indicate genes up-regulated in only one of the two *MBM8* lines. The colour scale in (**a**) and (**b**) represents log_2_fold changes of FPKM values. **c** Phylogenetic analysis of the protein sequences from (**a**) and (**b**) of overlapped DEGs up- or down-regulated in both *MBM8* and *MBM29* plants compared with *GUS* plants with the ARR proteins from Arabidopsis.

**
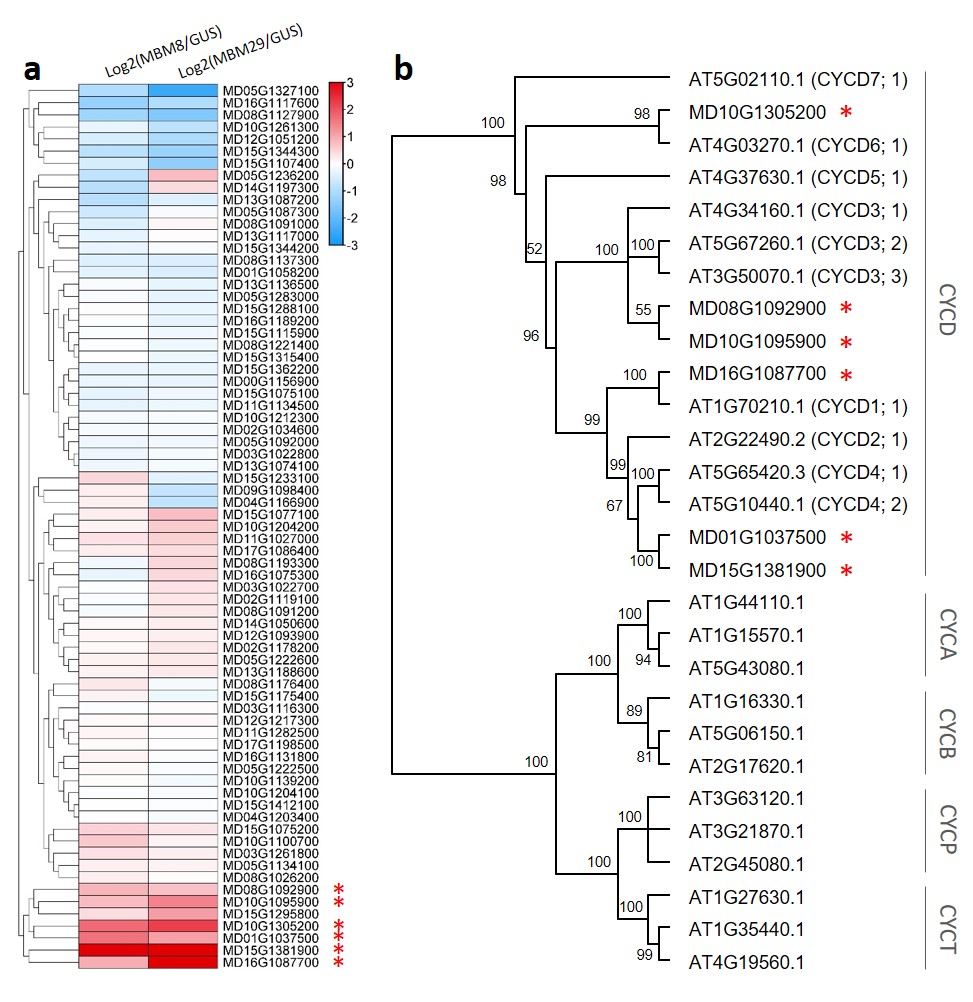
Supplemental Figure 9. Expression changes of D-class cyclin (*CYCD*) genes between *MBM* and *GUS* plants. a** Comparison of expression levels of apple cyclin genes between *MBM* and *GUS* plants. The genes marked by the red asterisks are *CYCD*s up-regulated in both *MBM8* and *MBM29*. The colour scale represents log_2_fold changes of FPKM values. **b** Phylogenetic analysis of the protein sequences of DEGs from (a) and cyclin proteins of Arabidopsis.

**
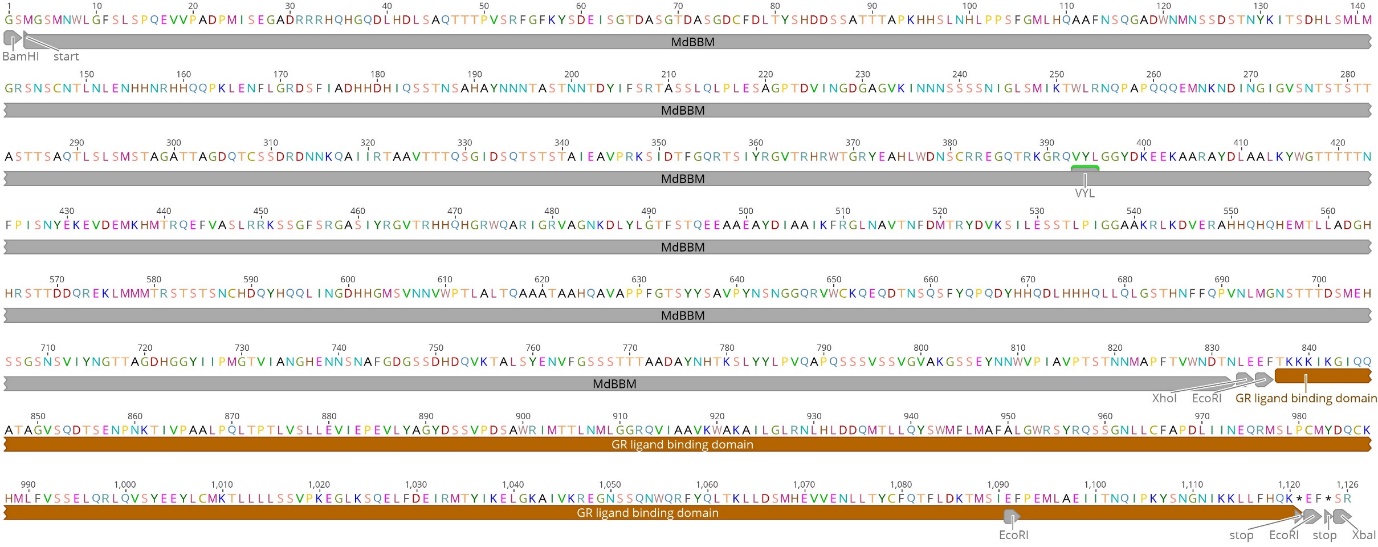
**

**Supplemental Figure 10 Diagram showing the design of the MdBBM1**-**GR translational fusion used to generate the plant transformation vector, pSAK778-MdBBM1-GR.**

**
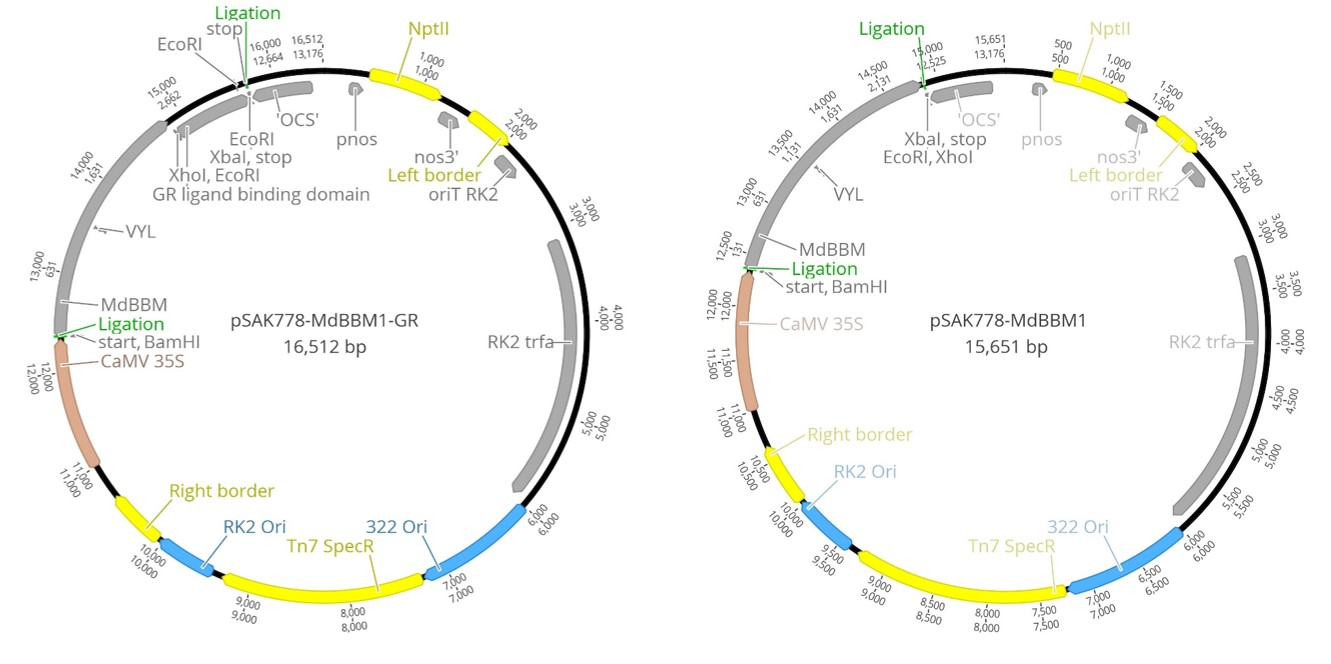
**

**Supplemental Figure 11 Maps of plant transformation vectors pSAK778**-**MdBBM1**-**GR and pSAK778**-**MdBBM1.**
